# Risk Assessment of Hazmat Road Transportation Considering Environmental Risk under Time-Varying Conditions

**DOI:** 10.3390/ijerph18189780

**Published:** 2021-09-17

**Authors:** Liping Liu, Qing Wu, Shuxia Li, Ying Li, Tijun Fan

**Affiliations:** School of Business, East China University of Science and Technology, Shanghai 200237, China; lpliu@ecust.edu.cn (L.L.); y30191399@mail.ecust.edu.cn (Q.W.); sxli@ecust.edu.cn (S.L.); liying@ecust.edu.cn (Y.L.)

**Keywords:** risk assessment, hazardous materials road transportation, environmental risk, population exposure risk, time-varying conditions

## Abstract

Hazardous materials shipments are integral to the development of industrial countries. Significant casualties and severe environmental pollution quickly ensue when accidents occur. Currently, relevant research on risk assessment of hazardous materials’ road transportation remains limited when both the population exposure risk and environmental risk are considered, especially in regard to analyzing the differences of accident impacts in different populations and environments. This paper adopts a Gaussian plume model to simulate dynamic areas at three levels of population exposure and assesses the pollution scope of air, groundwater, lakes, and rivers with a variety of diffusion models. Then, we utilize various costs to analyze the differences of accident impacts in population exposure and environmental pollution. Finally, a risk assessment model of hazardous materials road transportation under time-varying conditions is presented by considering the bearing capacity of the assessed area. Furthermore, this model is applied to a case study involving a risk assessment of hazardous materials transportation of a highly populated metropolitan area of Shanghai, China. The resulting analyses reveal that the safety of hazardous materials transportation could be effectively improved by controlling certain model parameters and avoiding road segments with a high risk of catastrophic accident consequences.

## 1. Introduction

Hazardous materials (hazmat) are flammable, explosive, toxic, corrosive and radioactive. Four billion tons of hazmat are transported worldwide, and approximately 400 million tons are shipped across China every year [1]. Hazmat accidents during road transport have caused catastrophic losses to humans and the environment worldwide. In July 2019, an oil truck in Nigeria lost control and turned over during driving, which caused heavy smog and led to 48 deaths and more than 90 injuries. In June 2020, a liquefied gas explosion accident occurred on an expressway in Zhejiang Province, China, causing 20 deaths and 172 hospitalizations. In September 2020, a truck carrying chlorine tanks exploded in Western Iran. The resulting explosion injured 217 people and released a large amount of chlorine into the air.

Therefore, risk assessment in hazmat transportation constitutes an important research area to enhance our understanding of hazmat transport management. In this paper, we analyze the differences in population exposure and environmental pollution due to accidents in different areas and propose a population exposure and environmental risk assessment model to better identify high-risk road segments, prevent the occurrence of catastrophic consequences, and provide a better guarantee for the safety of hazmat transportation.

The rest of this paper is organized as follows. Section 2 provides a literature review on hazmat transportation risk assessment and notes the differences between existing research and our contribution. Section 3 constructs the population exposure and environmental risk assessment model. The resulting analyses of the case study in Shanghai are presented in Section 4. Finally, conclusions are drawn in Section 5.

## 2. Literature Review

In previous studies, various risk assessment models have been presented to assess the potential risk of hazmat transportation.

In 1980, a traditional risk (TR) model was proposed [2]. Suppose a road network *G* = (*U*, *V*), consisting of a node set *U* to represent road interactions and a link set *V* to represent road segments; node *i*, *j* ϵ *U*, and segment (*i*, *j*) ϵ *V*. Then, the hazmat transportation risk *R* along a route is equal to the sum of the product of the accident probability *P_ij_* and accident consequence *C_ij_* of each road segment, as expressed in Equation (1).

(1) $$R=\sum_{(i,j)\in V}P_{ij}\times C_{ij}$$

Considering risk preference, Abkowitz et al. [3] proposed a perceived risk (PR) model by introducing risk preference parameter *q* to reflect the level of decision maker risk preference.

Then, Sivakumar et al. [4] proposed a conditional risk (CR) model, which evaluates the risk after the occurrence of the first hazmat accident. Erkut et al. [5] developed the minimum maximum (MM) model, mean-variance (MV) model, and disutility (DU) model.

Subsequently, Kang [6] and Kang et al. [7] applied the VaR risk measurement tool ubiquitous in financial risk management to hazmat shipments and proposed the value at risk (VaR) model to assess the risk of hazmat transportation. Toumazis et al. [8] improved the VaR model and developed the conditional value at risk (CVaR) model. The VaR model only focuses on the part of the risk not exceeding the VaR value, while the CVaR model focuses on the part of the risk exceeding the CVaR value [9].

Risk assessment under time-varying conditions is an emerging analysis topic to identify crash-prone traffic conditions and improve the safety of transportation. Wei [10] proposed a time-varying risk (TVR) model, which studies the change characteristics of accident probability and population density under time-varying conditions. Hossain et al. [11] applied a Bayesian belief net (BBN) to build a crash prediction model based on real-time traffic condition data retrieved from a high-resolution detector. Yu et al. [12] introduced a support vector machine (SVM) to evaluate real-time crash risk and analyze the relationship between risk and real-time traffic data detected by 30 RTMS radars. Sun et al. [13] proposed a dynamic Bayesian network (DBN) model of time sequence traffic data to investigate the relationship between crash occurrence and dynamic speed condition data. Wang et al. [14] presented a multilevel Bayesian logistic regression model of crash risk considering traffic, geometric and weather factors under time-varying conditions. Xu et al. [15] investigated the impacts of real-time traffic flow conditions on crash casualties under different collision types via geometric and traffic data. Yang et al. [16] introduced Bayesian dynamic logistic regression (Bayesian dynamic LR) to develop a real-time crash risk evaluation model with in-field streaming traffic data. Yu et al. [17] proposed a real-time crash risk analysis approach with multidimensional traffic flow input features to establish relationships between crash occurrence probability and precrash traffic operational conditions. Li et al. [18] built a real-time crash risk model of arterials with the long–short-term memory convolutional neural network (LSTM-CNN), which explicitly learns from various features, such as traffic flow characteristics, signal timing, and weather conditions.

In the risk assessment model of hazmat transportation, in addition to population exposure, environmental pollution should be considered [19].

On the one hand, most works in the literature have considered population exposure risk. Fabiano et al. [20] modified the accident probability of hazmat transportation taking into account the aspects of road characteristics, weather and traffic flow and analyzed the number of exposed people. Zhang et al. [21] applied the information diffusion theory to estimate the accident probability of hazardous chemical transportation and simulated the accident scope and number of people affected in liquefied petroleum gas leakage accidents at different levels. Fan et al. [22] adopted the product of the accident probability and number of affected populations within the determined scope along a given road as the risk. Zero et al. [23] associated the triangular fuzzy number (TFN) of population exposure in hazmat transportation accidents with the risk of the network. Dong et al. [24] and Sun [25] adopted a population exposure risk objective in their optimization model. In addition, Khanmohamadi et al. [26] and Orozco et al. [27] utilized ALOHA software to simulate the influence scope and identify the number of people exposed. Most studies have paid more attention to population. However, when assessing the accident consequences of population exposure, less consideration is given to the differences in the impact of accidents on populations in different areas.

On the other hand, there are few studies that consider both population exposure risk and environmental risk. Ren [28] and Xia et al. [29] analyzed the number of population exposures and the scope of environmental pollution. Saat et al. [30] estimated the environmental cleaning costs of soil and groundwater and the evacuation costs associated with population exposure caused by hazmat railway transportation accidents according to the accident scope specified in the Emergency Response Guidelines (ERG) of the United States. Cordeiro et al. [31] employed a multicriteria analysis method to assess accident consequences such as population vulnerability and environmental vulnerability and developed a risk assessment model. Huang et al. [32] examined the vulnerability of social and environmental functional areas along a road and established a risk assessment of the area along the highway (RAAH) model. Wang et al. [33] comprehensively calculated losses in terms of casualties and environmentally sensitive areas when assessing the time-varying risk of road segments. In addition, research on diffusion models and environmental bearing capacity has been relatively comprehensive in the field of environmental science. A diffusion model simulates the diffusion and migration of hazmat in the environment, so it can be adopted to evaluate the influence scope of hazmat transportation accidents. Zhao et al. [34] incorporated a Gaussian plume model into the box model to assess the air pollution area at facilities and routes. Yuan et al. [35] calculated the influence scope of the volatilization and diffusion of hazardous chemicals driven by wind with a Gaussian plume model. Zhao et al. [36] focused on three bodies of water, namely, rivers, lakes, and aquifers, and proposed a water diffusion model for each considering pipeline transportation accidents. The environmental bearing capacity is the natural purification capacity of the environment. Within the capacity limit, pollutants discharged into the environment may not cause harm to human health or the natural ecology through natural circulation [37]. Wang et al. [38] developed an evaluation index system for water environment carrying capacity (WECC) that considers the mutual interactions among six subsystems: industry, agriculture, population, water supply, water ecology, and water pollution. Peng et al. [39] built an indicator system of urban resources and environmental carrying capacity (URECC) based on ecological civilization, which included 18 indicators chosen from water, land, atmospheric environment, energy, and solid waste factors. Zhang et al. [40] included PM2.5 in their index system and established an evaluation system of regional resources and environmental carrying capacity (RECC). Thus, it is necessary to introduce the theory and method of environmental science into risk assessment of hazmat transportation. Moreover, most works in the literature consider the impact of hazmat accidents on a single environmental resource such as air or water, and seldom distinguish the impact of accidents on different environmental resources.

Furthermore, accident consequences may be affected by the bearing capacity of the assessed area. The bearing capacity of the assessed area includes the acceptable value of the population affected and the carrying capacity of the environment in the area. Within the capacity limit, the accident consequences may not cause serious casualties and harm to the natural ecology in the area. Although the accident consequences of a certain road segment are limited, they could be unacceptable if they exceed the bearing capacity range. In contrast, accident consequences could be significant, but could also be acceptable within the bearing capacity range. Hence, decision makers should appropriately process accident consequences according to the severity of the accident to better determine those road segments actually experiencing catastrophic consequences. More specifically, we set the population exposure risk preference parameter as higher than 1 if accident consequence exceeded the acceptable value of the population affected, or lower than 1 if it did not exceed it. We set the environmental pollution risk preference parameter as higher than 1 if accident consequence exceeded the carrying capacity of the environment, or lower than 1 if it did not exceed it.

Accounting for the above mentioned research gaps, this paper aims to develop a risk assessment model of hazmat road transportation under time-varying conditions. The main contributions of this paper can be summarized as follows:

(1) A Gaussian plume model was adopted to simulate the dynamic areas at three levels of population exposure under time-varying conditions and utilized evacuation costs, inspection costs, medical costs and casualties to analyze the difference in the impact of accidents on populations in different areas.
(2) We assessed the pollution scope of air, groundwater, lakes, and rivers with a variety of diffusion models under time-varying conditions and extended the pollution consequences to emergency disposal costs, monitoring costs and pollution remediation costs in order to scientifically evaluate the environmental pollution consequences of hazmat accidents.
(3) Population exposure and environmental pollution risk preference parameters were set according to the severity of population exposure, considering the acceptable upper limit of population exposure and the seriousness of environmental pollution, accounting for the environmental bearing capacity. Then, population exposure and environmental risk assessment models were built under time-varying conditions.

Therefore, this paper improves the risk analysis system of hazmat transportation under time-varying conditions and provides a solid foundation for research on hazmat transportation location and path optimization.

## 3. Model

### 3.1. Leakage Accident Probability Model under Time-Varying Conditions

In this paper, Table 1 lists the weighted averages of truck accident probabilities in three major states determined by Harwood et al. [41]. We regard them as the initial truck accident probability $P_{ij}^{A}$. Table 2 shows the conditional leakage accident probability $P_{ij}^{B}$ under the given accident types. Then, the product of $P_{ij}^{A}$ and $P_{ij}^{B}$ is applied to express the leakage accident probability $P_{ij}^{C}$ [28]. In other words, $P_{ij}^{A}$ and $P_{ij}^{B}$ are multiplied to obtain $P_{ij}^{C}$.

Therefore, the leakage accident probability of hazmat road transportation is expressed in Equation (2). For the convenience of follow-up research, the unit is converted as indicated in Equation (3), where *P_ij_*(*t*) is the leakage accident probability in road segment (*i*, *j*) at time *t*; *M_ij_*(*t*) is the vehicle density of road segment (*i*, *j*) at time *t*, vehicle/km; and *L_ij_* is the driving length of road segment (*i*, *j*), km.

(2) $$P_{ij}^{C}=P_{ij}^{A}\times P_{ij}^{B}$$

(3) $$P_{ij}\left(t\right)=10^{-6}\times P_{ij}^{A}\times P_{ij}^{B}\times M_{ij}\left(t\right)\times L_{ij}{}^{2}$$

### 3.2. Accident Consequence Assessment Model under Time-Varying Conditions

In this paper, the following assumptions apply when establishing the accident consequence assessment model: (1) hazmat immediately leaks after the vehicle overturns on the road, and the vehicle is regarded as a point risk source, where pollution spreads along all directions simultaneously into the external environment [2,34]; (2) the spilled hazmat does not degrade via the process of diffusion and spreads into air and water resources in equal amounts; (3) leakage occurs after an accident in a road segment, which does not affect the population and environment along other road segments.

#### 3.2.1. Accident Consequence Model of Population Exposure

In this paper, a Gaussian plume model is applied to simulate the accident influence area of population exposure. The Gaussian plume model simulates the diffusion process of hazmat into air, so the impact area of air pollution simulated with this model is also applicable to determine the population exposed to air at any time.

Based on relevant research on the Gaussian plume model in references [34,35], the initial model is as follows:

(4) $$C_{ij}(x,y,z,H,t)=\frac{Q^{Hazard}}{\pi v_{ij}^{Wind}(t)\sigma_{y}\sigma_{z}}\times \exp\left(-\frac{y^{2}}{2\sigma_{y}{}^{2}}\right)\times \left\{\exp\left[-\frac{{\left(z-H\right)}^{2}}{2\sigma_{z}{}^{2}}\right]+\exp\left[-\frac{{\left(z+H\right)}^{2}}{2\sigma_{z}{}^{2}}\right]\right\}$$

where *C_ij_*(*x*, *y*, *z*, *H*, *t*) is the concentration of hazmat at downwind point (*x*, *y*, z) in road segment (*i*, *j*) at time *t*, mg/m^3^; *x*, *y* and *z* are the transversal distance, longitudinal distance and vertical distance, respectively, between the measuring point and leakage source in the case of downwind diffusion, m; *H* is the height of the leakage source, m; *Q^Hazard^* is the release speed of hazmat from the leakage source, mg/s; $v_{ij}^{Wind}(t)$ is the wind speed along road segment (*i*, *j*) at time *t*, m/s; *σ_y_* is the horizontal diffusion coefficient, m; and *σ_z_* is the vertical diffusion coefficient, m.

In this model, *σ_y_* and *σ_z_* mainly depend on the atmospheric stability parameters *a*, *b*, *c*, *d* and *x* [42], where *σ_y_* = *ax^c^* and *σ_y_* = *bx^d^*. Suppose that *y*, *z*, and *H* are not considered, the initial model is modified as follows:

(5) $$C_{ij}(x,t)=\frac{Q^{Hazard}}{\pi abv_{ij}^{Wind}(t)x^{c+d}}$$

**Figure 1:**
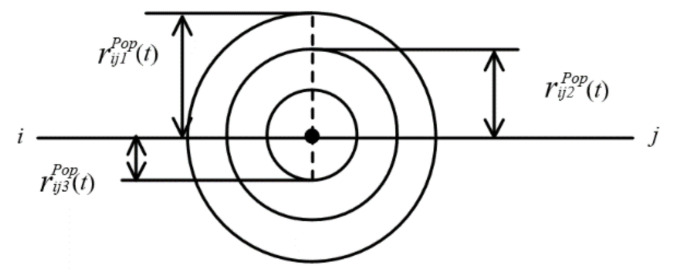
Accident influence area of population exposure.

According to the three injury threshold levels of the AEGL standard, three regions are divided, which are denoted by *u* = 1, 2, and 3. $C_{u}^{Pop}$ is set as the limit value of the hazmat concentration in region *u*, mg/m^3^. The radius $r_{iju}^{Pop}(t)$ of population exposure due to the point leakage source in road segment (*i*, *j*) at time *t* is expressed in Equation (6), and the accident influence area $S_{iju}^{Pop}(t)$ of population exposure along road segment (*i*, *j*) is shown in Figure 1 and Equation (7), where $r_{ij4}^{Pop}(t)$ = 0. $S_{iju}^{Pop}(t)$ is a circle or ring based on the above assumption (1).

(6) $$r_{iju}^{Pop}(t)={\left(\frac{Q^{Hazard}}{\pi abv_{ij}^{Wind}(t)C_{u}^{Pop}}\right)}^{\frac{1}{c+d}}$$

(7) $$S_{iju}^{Pop}(t)=\pi \left\{{\left[\frac{Q^{Hazard}}{\pi abv_{ij}^{Wind}(t)C_{u}^{Pop}}\right]}^{\frac{2}{c+d}}-{\left[\frac{Q^{Hazard}}{\pi abv_{ij}^{Wind}(t)C_{u+1}^{Pop}}\right]}^{\frac{2}{c+d}}\right\}$$

AEGL standards [43] were developed by the National Advisory Committee for Acute Exposure Guideline Levels for Hazmat (NAC/AEGL Committee) and recommended to the Environmental Protection Agency (EPA). In this paper, the AEGL standard is adopted as the $C_{u}^{Pop}$ value, and Table 3 summarizes the classification of AEGL injury threshold levels.

Considering the differences in the population affected by accidents, this paper divides the costs of population exposure into four parts: evacuation costs, inspection costs, medical costs and losses associated with casualties. The four parts are expressed as $F_{v}^{Pop}$, where *v* = 1, 2, 3, and 4, including 1—evacuation costs; 2—inspection costs; 3—medical costs; and 4—losses associated with casualties. Evacuation costs can be converted into costs associated with the mobilization of medical, security police, traffic police and fire control departments. Inspection costs include internal and external examination costs, routine blood analysis costs, routine urine analysis costs, chest X-ray costs and electrocardiogram costs. Medical costs comprise the costs of registration, examination, laboratory testing, diagnosis, medication, surgery, hospitalization and rescue. The losses associated with casualties encompass relevant compensations.

*T_ij_*(*t*) is the population density along road segment (*i*, *j*) in area *u* at time *t*. The probability of cost type *v* generated by population exposure is *α_uv_*(*t*). Therefore, the total accident consequence of population exposure along road segment (*i*, *j*) under time-varying conditions is defined in Equation (8).

(8) $$C_{ij}^{Pop}(t)=\sum_{u=1}^{3}\sum_{v=1}^{4}S_{iju}^{Pop}(t)\times T_{ij}(t)\times \alpha_{uv}(t)\times F_{v}^{Pop}$$

#### 3.2.2. Accident Consequence Model of Environmental Pollution

In this paper, the concentrations, influence lengths and areas of the various kinds of environmental pollution caused by accidents in road segment (*i*, *j*) at time *t* are expressed as $C_{r}^{Env}$, $r_{ijr}^{Env}(t)$ and $V_{ijr}^{Env}(t)$, respectively, where *r* = 1, 2, 3, and 4, for 1—air; 2—groundwater; 3—lakes; and 4—rivers.

**Figure 2:**
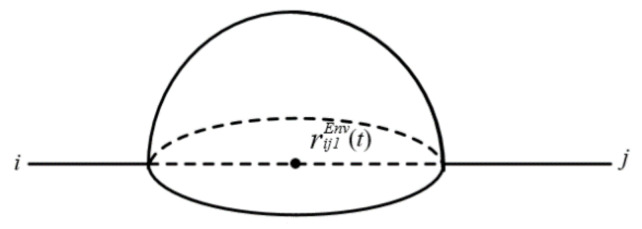
Accident influence area of air pollution.

We assessed the air pollution area. $C_{1}^{Env}$ is the limit value of the hazmat concentration in air (refers to the AEGL-2 standard). Based on Equation (6), the radius of the air pollution influence area of a given point leakage source in road segment (*i*, *j*) at time *t* is expressed in Equation (9). The accident influence area of air pollution along road segment (*i*, *j*) is shown in Figure 2 and Equation (10), which is a hemisphere [34].

(9) $$r_{ij1}^{Env}(t)={\left(\frac{Q^{Hazard}}{\pi abv_{ij}^{Wind}(t)C_{1}^{Env}}\right)}^{\frac{1}{c+d}}$$

(10) $$V_{ij1}^{Env}(t)=\frac{2}{3}\pi {\left[\frac{Q^{Hazard}}{\pi abv_{ij}^{Wind}(t)C_{1}^{Env}}\right]}^{\frac{3}{c+d}}$$

Then, we simulated the water resource pollution area. In this paper, water resources are divided into groundwater and surface water, and surface water can be divided into static surface water (lakes) and dynamic surface water (rivers).

**Figure 3:**
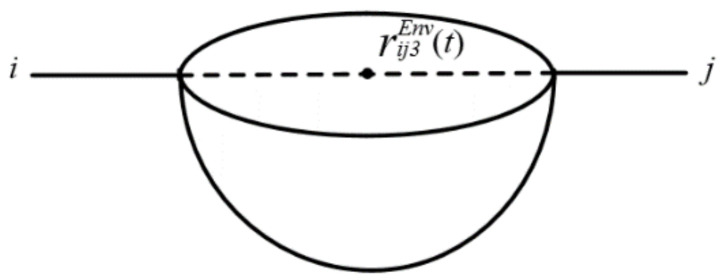
Accident influence area of groundwater pollution.

First, $C_{2}^{Env}$ is the limit value of the hazmat concentration in groundwater (refer to [45], the same below). Based on the groundwater diffusion model presented in [36], the influence radius of groundwater pollution due to the point leakage source in road segment (*i*, *j*) at time *t* is expressed in Equation (11), and the accident influence area (hemisphere) of groundwater along road segment (*i*, *j*) is shown in Figure 3 and Equation (12).

(11) $$r_{ij2}^{Env}(t)=v_{ij}^{Gwater}(t)t^{Gwater}+\sqrt{H_{ij}^{Gwater}u^{*}t^{Gwater}\times \ln\left(\frac{Q}{C_{2}^{Env}S_{ij}^{Gwater}\sqrt{\pi H_{ij}^{Gwater}u^{*}t^{Gwater}}}\right)}$$

where $v_{ij}^{Gwater}(t)$ is the average flow velocity of groundwater along road segment (*i*, *j*) at time *t*, m/s; *t^Gwater^* is the time after hazmat release into groundwater, s; $H_{ij}^{Gwater}$ is the average depth of groundwater along road segment (*i*, *j*), m; u* is the friction velocity of water flow [46], m/s, with the assumed value occurring in the interval of [0.02, 0.553]; *Q* is the release amount of hazmat into water, mg; and $S_{ij}^{Gwater}$ is the wet cross-sectional area of groundwater along road segment (*i*, *j*), m^2^.

(12) $$V_{ij2}^{Env}(t)=\frac{2}{3}\pi {\left[v_{ij}^{Gwater}(t)t^{Gwater}+\sqrt{H_{ij}^{Gwater}u^{*}t^{Gwater}\times \ln\left(\frac{Q}{C_{2}^{Env}S_{ij}^{Gwater}\sqrt{\pi H_{ij}^{Gwater}u^{*}t^{Gwater}}}\right)}\right]}^{3}$$

Second, $C_{3}^{Env}$ is the limit value of the hazmat concentration in lake water. According to the lake diffusion model reported in [36], the lake pollution influence radius of the point leakage source in road segment (*i*, *j*) at time *t* is defined in Equation (13).

(13) $$r_{ij3}^{Env}(t)=\sqrt{0.58H_{ij}^{Lake}u^{*}t^{Lake}\times \ln\left(\frac{Q}{0.58\pi H_{ij}^{Lake}{}^{{}^{2}}u^{*}t^{Lake}C_{3}^{Env}}\right)}$$

where *t^Lake^* is the diffusion time of hazmat into lake water, s; and $H_{ij}^{Lake}$ is the average depth of the lake along road segment (*i*, *j*), m.

Third, $C_{4}^{Env}$ is the limit value of the hazmat concentration in river water. Based on the river diffusion model proposed in [36], the influence radius of lake pollution of the point leakage source in road segment (*i*, *j*) at time *t* is expressed in Equation (14).

(14) $$r_{ij4}^{Env}(t)=v_{ij}^{River}(t)t^{River}+\sqrt{0.58H_{ij}^{River}u^{*}t^{River}\times \ln\frac{Q\left[2+\exp\left(-\frac{W_{ij}^{River}{}^{{}^{2}}}{0.145H_{ij}^{River}u^{*}t^{River}}\right)\right]}{0.58\pi H_{ij}^{River}{}^{{}^{2}}u^{*}t^{River}C_{4}^{Env}}}$$

where $v_{ij}^{River}(t)$ is the longitudinal velocity of river flow along road segment (*i*, *j*) at time *t*, m/s; $t^{River}$ is the time after hazmat release into the river, s; $H_{ij}^{River}$ is the average depth of the river along road segment (*i*, *j*), m; and $W_{ij}^{River}$ is the average width of the river along road segment (*i*, *j*), m.

**Figure 4:**
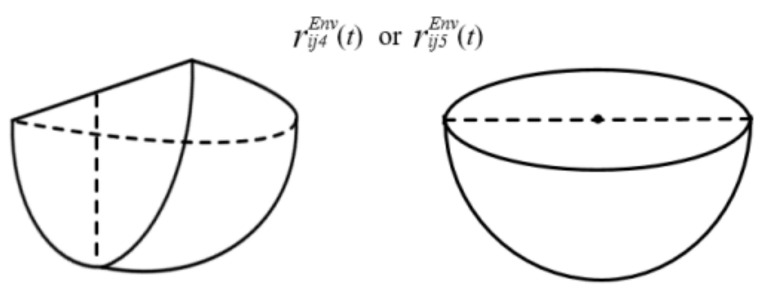
Accident influence area of lake and river pollution.

Then, the accident influence area of lake and river pollution can be divided into two situations. One situation indicates that road segment (*i*, *j*) occurs along the lake or river, and the influence area (one-fourth of a sphere) is shown in Figure 4(left). The other situation states that road segment (*i*, *j*) passes through the lake or river, and the influence area (hemisphere) is shown in Figure 4(right) [36]. Then, the influence area (hemisphere) of lake and river pollution along road segment (*i*, *j*) is expressed in Equations (15) and (16), respectively.

(15) $$V_{ij3}^{Env}(t)=\left(\frac{1}{3}or\frac{2}{3}\right)\pi {\left[0.58H_{ij}^{Lake}u^{*}t^{Lake}\times \ln\left(\frac{Q}{0.58\pi H_{ij}^{Lake}{}^{{}^{2}}u^{*}t^{Lake}C_{3}^{Env}}\right)\right]}^{\frac{3}{2}}$$

(16) $$V_{ij4}^{Env}(t)=\left(\frac{1}{3}or\frac{2}{3}\right)\pi {\left\{v_{ij}^{River}(t)t^{River}+\sqrt{0.58H_{ij}^{River}u^{*}t^{River}\times \ln\frac{Q\left[2+\exp\left(-\frac{W_{ij}^{River}{}^{{}^{2}}}{0.145H_{ij}^{River}u^{*}t^{River}}\right)\right]}{0.58\pi H_{ij}^{River}{}^{{}^{2}}u^{*}t^{River}C_{4}^{Env}}}\right\}}^{3}$$

In this paper, the pollution of air and water resources (groundwater, lakes and rivers) caused by accidents is comprehensively considered. According to the Recommended Method of Environmental Damage Appraisal and Assessment (version II) issued by the Ministry of Ecology and Environment of China (http://www.mee.gov.cn/gkml/hbb/bgt/201411/W020141105395741560668.pdf, accessed on 5 October 2020), the environmental pollution treatment costs associated with accidents in each road segment are divided into emergency disposal costs, monitoring costs and pollution remediation costs. These costs are expressed as $F_{rw}^{Env}$, where *r* = 1, 2, 3, and 4, for 1—air pollution, 2—groundwater pollution, 3—lake pollution, and 4—river pollution; *w* = 1, 2, and 3, for 1—emergency disposal costs, 2—monitoring costs, and 3—pollution remediation costs. Emergency disposal costs include pollution control costs, on-site rescue costs, site cleaning costs, personnel transfer and resettlement costs, and emergency monitoring costs. Monitoring costs consist of the expenses for environmental monitoring, information disclosure, on-site investigation and supervision implementation. Pollution remediation costs comprise the expenses of scheme preparation and remediation and subsequent monitoring and supervision.

Therefore, the total consequence of environmental pollution in road segment (*i*, *j*) under time-varying conditions is as follows:

(17) $$C_{ij}^{Env}(t)=\sum_{r=1}^{4}\sum_{w=1}^{3}V_{ijr}^{Env}(t)\times F_{rw}^{Env}$$

### 3.3. Transportation Risk Assessment Model under Time-Varying Conditions

#### 3.3.1. Population Exposure Risk Assessment Model

In this paper, the risk preference parameter $q_{ij}^{Pop}$ in the population exposure risk model is set according to the ratio $p_{ij}^{Pop}$ of the actual population affected by the accident to the acceptable upper limit of the population affected along segment (*i*, *j*). This parameter is related to the severity of the accident impact on the population, which is set as follows:

(18) $$p_{ij}^{Pop}=\frac{\sum_{u=1}^{3}S_{iju}^{Pop}(t)\times T_{ij}(t)}{10^{3}\times L_{ij}\times d^{Pop}\times T_{ij}(t)}$$

where *d^P^^op^* is the width of the bearing area (referring to 600 m, as specified in the Emergency Response Guide [47]), m.

(19) $$q_{ij}^{Pop}=\left\{\begin{array}{cc} 0.9, & 0<p_{ij}^{Pop}\leq 0.5\\ 0.95, & 0.5<p_{ij}^{Pop}<1\\ 1.0 & p_{ij}^{Pop}=1\\ 1.05 & 1<p_{ij}^{Pop}\leq 1.5\\ 1.1 & p_{ij}^{Pop}>1.5 \end{array}\right.$$

Therefore, based on the leakage accident probability and accident consequence models of population exposure, the population exposure risk assessment model of hazmat road transportation under time-varying conditions is as follows:

(20) $$ER_{ij}^{Pop}(t)=P_{ij}(t)\times C_{ij}^{Pop}{\left(t\right)}^{q_{ij}^{Pop}}$$

where $P_{ij}\left(t\right)=10^{-6}\times P_{ij}^{A}\times P_{ij}^{B}\times M_{ij}\left(t\right)\times L_{ij}{}^{2}$

(21) $$\begin{array}{ll} C_{ij}^{Pop}(t) & =\sum_{u=1}^{3}\sum_{v=1}^{4}S_{iju}^{Pop}(t)\times T_{ij}(t)\times \alpha_{uv}(t)\times F_{v}^{Pop}\\ & =\sum_{v=1}^{4}\pi \times \left\{{\left[\frac{Q^{Hazard}}{\pi abv_{ij}^{Wind}(t)C_{1}^{Pop}}\right]}^{\frac{2}{c+d}}-{\left[\frac{Q^{Hazard}}{\pi abv_{ij}^{Wind}(t)C_{2}^{Pop}}\right]}^{\frac{2}{c+d}}\right\}\times \alpha_{1v}(t)\times T_{ij}(t)\times F_{v}^{Pop}\\ & +\pi \times \left\{{\left[\frac{Q^{Hazard}}{\pi abv_{ij}^{Wind}(t)C_{2}^{Pop}}\right]}^{\frac{2}{c+d}}-{\left[\frac{Q^{Hazard}}{\pi abv_{ij}^{Wind}(t)C_{3}^{Pop}}\right]}^{\frac{2}{c+d}}\right\}\times \alpha_{2v}(t)\times T_{ij}(t)\times F_{v}^{Pop}\\ & +\pi \times {\left[\frac{Q^{Hazard}}{\pi abv_{ij}^{Wind}(t)C_{3}^{Pop}}\right]}^{\frac{2}{c+d}}\times \alpha_{3v}(t)\times T_{ij}(t)\times F_{v}^{Pop} \end{array}$$

#### 3.3.2. Environmental Risk Assessment Model

In this paper, the risk preference parameter $q_{ij}^{Env}$ in the environmental risk model is introduced according to the ratio $p_{ij}^{Env}$ of the hazmat leakage amount in road segment (*i*, *j*) to the environmental bearing capacity of the area. This parameter is related to the severity of environmental pollution caused by the accident, as follows:

(22) $$p_{ij}^{Env}=\frac{Q}{\sum_{r=1}^{5}V_{ijr}^{Env}(t)\times C_{r}^{Env}}$$

(23) $$q_{ij}^{Env}=\left\{\begin{array}{cc} 0.9, & 0<p_{ij}^{Env}\leq 0.5\\ 0.95, & 0.5<p_{ij}^{Env}<1\\ 1.0 & p_{ij}^{Env}=1\\ 1.05 & 1<p_{ij}^{Env}\leq 1.5\\ 1.1 & p_{ij}^{Env}>1.5 \end{array}\right.$$

Therefore, based on the leakage accident probability model and accident consequence model of environmental pollution, the environmental risk assessment model of hazmat road transportation under time-varying conditions is expressed in Equation (23).

(24) $$ER_{ij}^{Env}(t)=P_{ij}(t)\times C_{ij}^{Env}{\left(t\right)}^{q_{ij}^{Env}}$$

where $P_{ij}\left(t\right)=10^{-6}\times P_{ij}^{A}\times P_{ij}^{B}\times M_{ij}\left(t\right)\times L_{ij}{}^{2}$

$$\begin{array}{ll} C_{ij}^{Env}(t) & =\sum_{r=1}^{5}\sum_{w=1}^{3}V_{ijr}^{Env}(t)\times F_{rw}^{Env}\\ & =\sum_{w=1}^{3}\frac{2}{3}\pi {\left[\frac{Q^{Hazard}}{\pi abv_{ij}^{Wind}(t)C_{1}^{Env}}\right]}^{\frac{3}{c+d}}\times F_{1w}^{Env}\\ & +\frac{2}{3}\pi {\left[v_{ij}^{Gwater}(t)t^{Gwater}+\sqrt{H_{ij}^{Gwater}u^{*}t^{Gwater}\times \ln\left(\frac{Q}{C_{2}^{Env}S_{ij}^{Gwater}\sqrt{\pi H_{ij}^{Gwater}u^{*}t^{Gwater}}}\right)}\right]}^{3}\times F_{2w}^{Env}\\ & +\left(\frac{1}{3}or\frac{2}{3}\right)\pi {\left[0.58H_{ij}^{Lake}u^{*}t^{Lake}\times \ln\left(\frac{Q}{0.58\pi H_{ij}^{Lake}{}^{{}^{2}}u^{*}t^{Lake}C_{3}^{Env}}\right)\right]}^{\frac{3}{2}}\times F_{3w}^{Env}\\ & +\left(\frac{1}{3}or\frac{2}{3}\right)\pi {\left\{v_{ij}^{River}(t)t^{River}+\sqrt{0.58H_{ij}^{River}u^{*}t^{River}\times \ln\frac{Q\left[2+\exp\left(-\frac{W_{ij}^{River}{}^{{}^{2}}}{0.145H_{ij}^{River}u^{*}t^{River}}\right)\right]}{0.58\pi H_{ij}^{River}{}^{{}^{2}}u^{*}t^{River}C_{4}^{Env}}}\right\}}^{3}\times F_{4w}^{Env} \end{array}$$

## 4. Case Study

**Figure 5:**
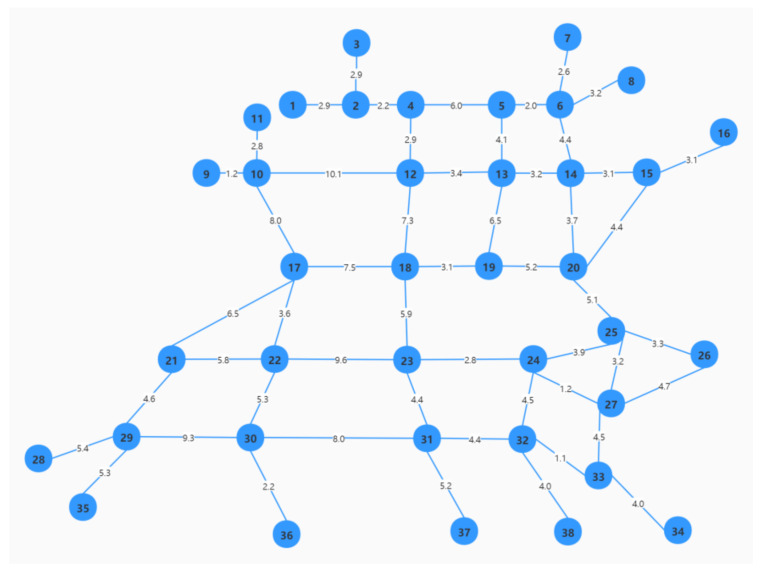
Road network in Songjiang District (simplified).

Shanghai is one of the most highly populated metropolitan cities in the world, and it experiences heavy traffic, especially during the morning and afternoon rush hours. We apply the proposed models to the newly constructed real-world transportation network of the district of Songjiang, Shanghai. The network is simplified and contains 38 nodes and 52 arcs, as shown in Figure 5. We obtained the lengths of the arcs according to Baidu maps and retrieved the basic traffic flows along the arcs from the Shanghai Public Data Open Platform (https://data.sh.gov.cn/, accessed on 6 March 2021). The traffic flow divided by the length is the traffic density.

Population density information was acquired from the 2020 Shanghai Statistical Yearbook issued by the Municipal Bureau of Statistics in Shanghai. We collected wind speed data in different periods of Songjiang from the Shanghai Meteorological Service (http://sh.cma.gov.cn/, accessed on 7 March 2021). The costs of population exposure are based on the price standards of medical services in Shanghai (https://max.book118.com/html/2018/1009/5313144021001321.shtm, accessed on 7 March 2021) and the Interpretation of Several Issues Concerning the Application of Law in the Trial of Personal Injury Compensation Cases (http://www.court.gov.cn/fabu-xiangqing-282621.html, accessed on 7 March 2021). The calculation of the environmental pollution treatment costs refers to the instructions in the Recommended Method of Environmental Damage Appraisal and Assessment (http://www.mee.gov.cn/gkml/hbb/bgt/201411/W020141105395741560668.pdf, accessed on 8 March 2021).

A truck transporting 6 tons of ammonia (gaseous) is considered in the network. The results of the case study are analyzed.

### 4.1. Analysis of the Accident Probability

**Figure 6:**
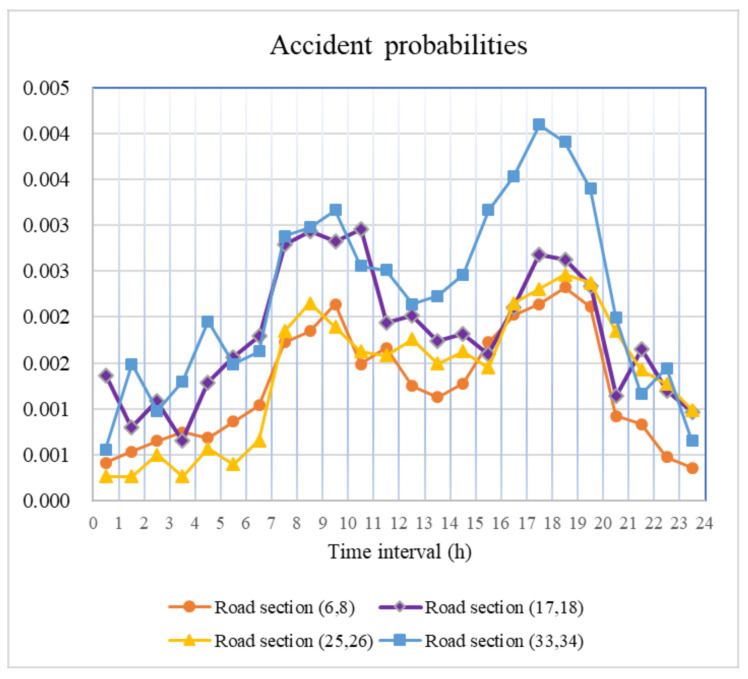
Accident probabilities.

Figure 6 shows the accident probabilities during 24 periods in road segments (6, 8), (17, 18), (25, 26) and (33, 34). It is clear that the accident probabilities of hazmat transportation are low, and the accident probabilities increase during the morning and afternoon rush hours but decrease during other periods (especially late at night).

**Figure 7:**
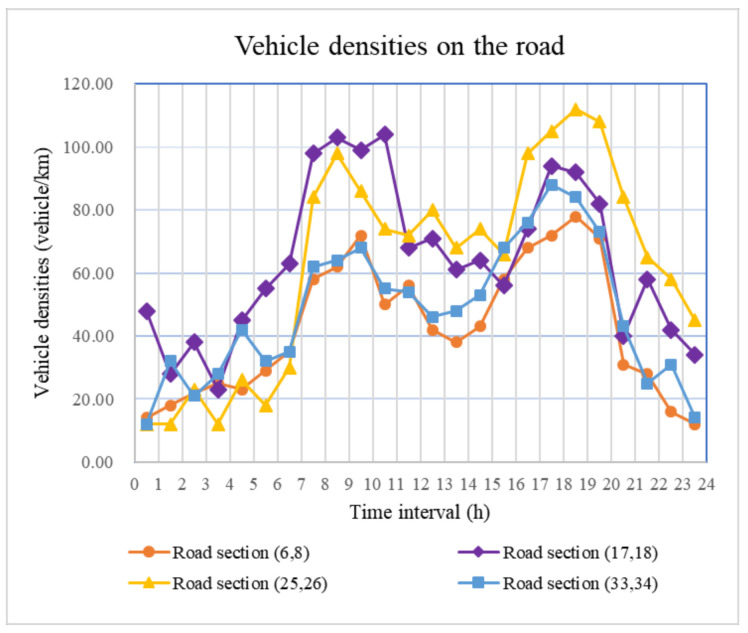
Vehicle densities on the roads.

For the same road segment, only the vehicle density *M_ij_*(*t*) in Equation (3) is affected by time-varying conditions. As vehicle densities on roads tend to increase during the morning and afternoon rush hours (Figure 7), the accident probabilities also exhibit a similar trend. Hence, we can effectively reduce accident probability by controlling vehicle density in practice.

### 4.2. Analysis of the Accident Consequences

**Figure 8:**
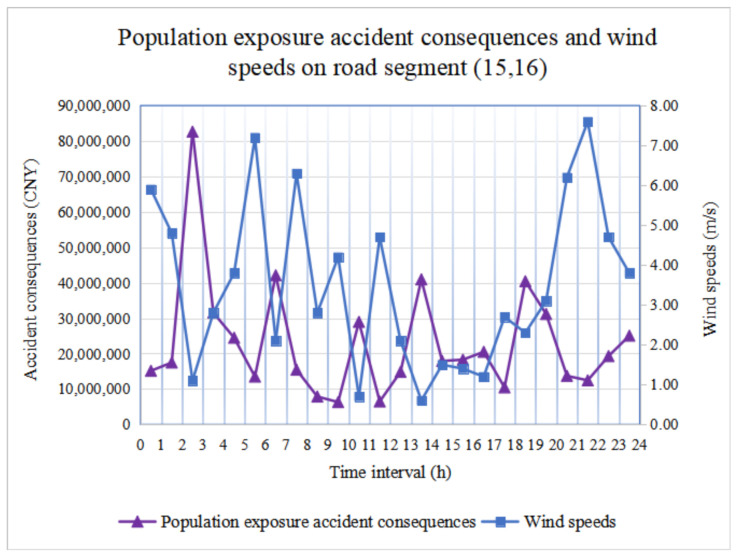
Population exposure accident consequences and wind speed in road segment (15, 16).

**Figure 9:**
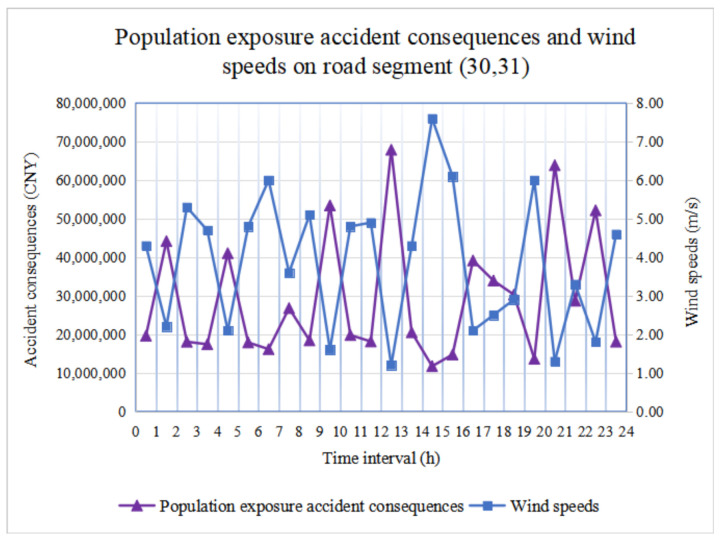
Population exposure accident consequences and wind speed in road segment (30, 31).

We determine population exposure accident consequences and wind speeds during 24 periods in road segments (15, 16) and (30, 31), as shown in Figure 8 and Figure 9, respectively. It is observed that there are significant population exposure accident consequences during hazmat transportation. In addition, for the same road segment, if *α_uv_*(*t*) remains fixed, only the wind speed $v_{ij}^{Wind}(t)$ and population density *T_ij_*(*t*) in Equation (7) are affected by time-varying conditions.

Although we simulate changes in population density in residential areas (population density is lower during the daytime and higher at night), commercial office areas (population density is higher during the daytime and lower at night), and boarding schools (population density during the daytime and at night is similar), the impact on population exposure accident consequences is limited and irregular.

However, Figure 8 and Figure 9 show that wind speed is inversely proportional to accident consequences. In other words, the higher the wind speed, the less significant the accident consequence is. Suppose that the diffusion time is not considered, and the amount of leaked hazmat remains the same; the higher the wind speed, the faster the diffusion process of hazmat occurs, which leads to the phenomenon of hazmat dilution. Because we set the hazmat concentration (referring to the AEGL standard) in advance, the influence area reaching the specified concentration is smaller, and the accident consequence is less remarkable. In practice, we should consider wind speed to avoid any catastrophic accident consequences for population exposure.

**Figure 10:**
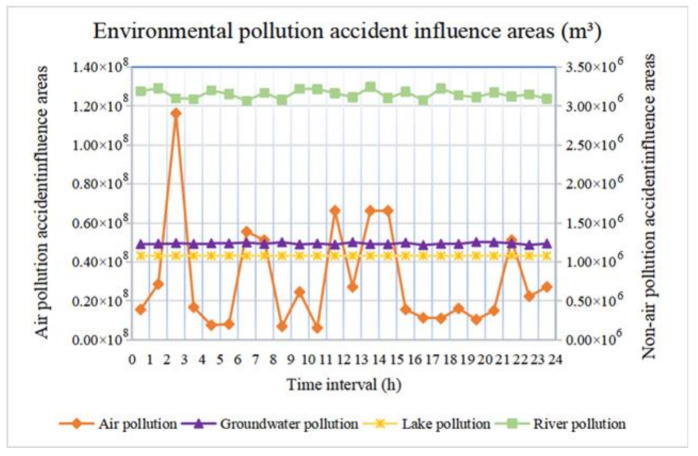
Environmental pollution accident influence areas.

Then, we determine the accident influence areas of air, groundwater, lake and river pollution during 24 periods along road segment (17, 18), as shown in Figure 10. The variation in the accident influence area of environmental pollution follows the order of air > river > groundwater > lake. Under time-varying conditions, only the wind speed $v_{ij}^{Wind}(t)$ in Equation (10) is affected. Equation (15) indicates that lakes remain motionless, so the accident influence area of lake pollution is independent of time. Then, under time-varying conditions, only the wind speed $v_{ij}^{Wind}(t)$ in Equation (10) is affected. Additionally, in Equations (12) and (16), $v_{ij}^{Gwater}(t)$ and $v_{ij}^{River}(t)$ are affected by time-varying conditions, but they are much lower than the wind speed, and the former is lower than the latter. The lower the speed, the smaller the variation difference is. Thus, the variation area of the accident influence area of groundwater pollution is smaller than that of river pollution and is obviously smaller than that of air pollution. Therefore, we should pay attention to the impact of time-varying conditions on the influence area of air pollution in practice.

**Figure 11:**
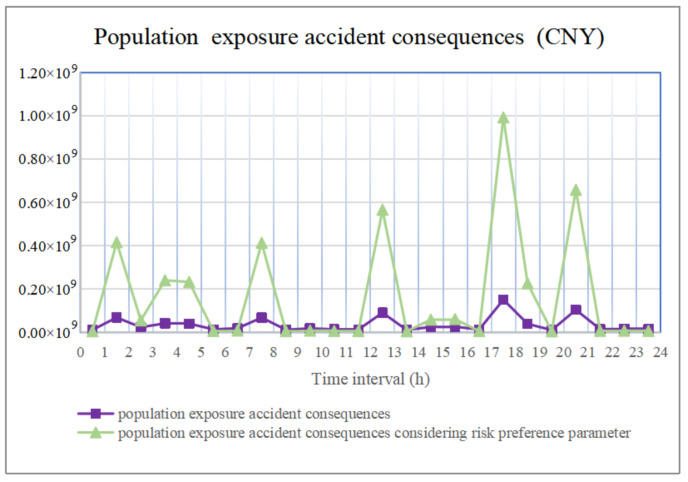
Population exposure accident consequences.

**Figure 12:**
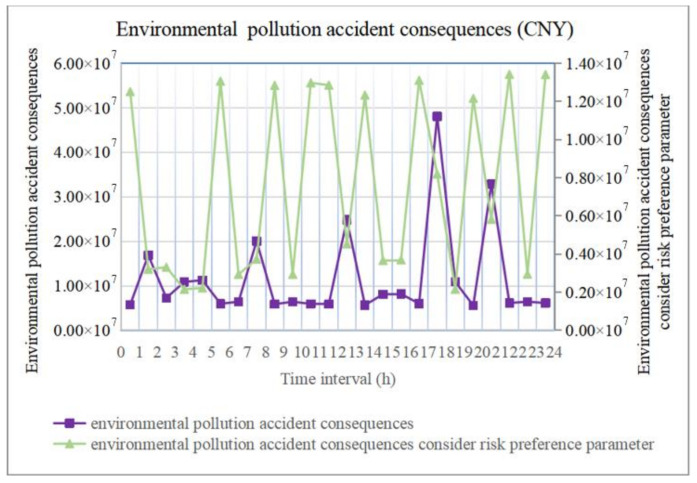
Environmental pollution accident consequences.

In addition, choosing road segment (32, 33) as an example, the accident consequences during 24 periods are shown in Figure 11 and Figure 12. The accident consequences of environmental pollution are similar to those of population exposure, and they are also quite notable. Hence, hazmat transportation accidents exhibit the characteristics of low probability and significant consequences.

In terms of population exposure and environmental pollution accident consequences, based on Equations (18), (19), (21) and (22), we set the population exposure risk preference parameter $q_{ij}^{Pop}$ as higher than 1 if accident consequence exceeded the acceptable value of the population affected, or lower than 1 if it did not exceed it; then, we set the environmental pollution risk preference parameter $q_{ij}^{Env}$ as higher than 1 if accident consequence exceeded the carrying capacity of the environment, or lower than 1 if it did not exceed it. This extends the consequences beyond the bearing capacity or reduces the consequences within the bearing capacity, respectively, as shown in Figure 11 and Figure 12, which clearly reveal the period when the accident actually causes catastrophic consequences. In addition, if we analyze the accident consequences of each road segment, the insights are the same.

### 4.3. Analysis of the Transportation Risk

**Figure 13:**
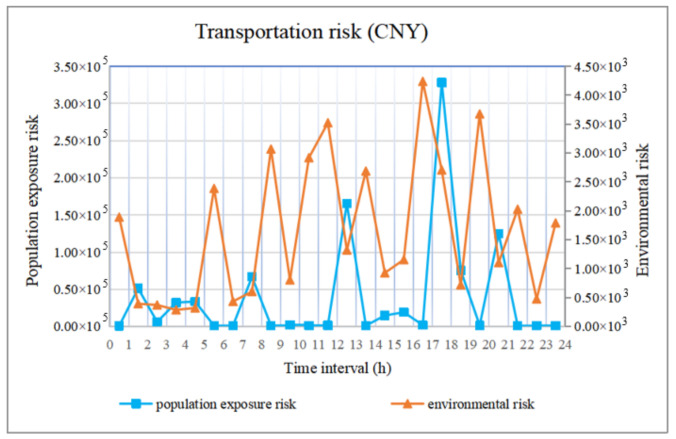
Transportation risk.

Comparing Figure 12 and Figure 13, the accident consequences considering the risk preference parameter and risk reveal the same trend during each period, and the corresponding transportation risks in the road segments with major accident consequences are higher. Because the risk preference parameter enhances the accident consequences to encompass the periods and road segments with serious accidents, this avoids the problem that certain periods and road segments with catastrophic accident consequences are overlooked due to a low probability when calculating risk.

When planning transportation routes or schemes, we should avoid segments with a higher transportation risk, and at the same time, we should reasonably avoid road segments with more notable accident consequences according to the characteristics of low probability and significant consequences in hazmat transportation accidents. In other words, we should consider transportation risk and avoid catastrophic consequences to identify road segments with a high risk of catastrophic consequences and improve the security of hazmat transportation.

## 5. Conclusions

Road accidents involving hazmat transportation have caused notable casualties and excessive environmental pollution. Therefore, it is necessary to avoid high-risk road segments and prevent catastrophic accidents through in-depth research and assessment of hazmat transportation risk. Based on the probability of leakage accidents, this paper explores transportation risk in two aspects of population exposure and environmental pollution and establishes a transportation risk assessment model under time-varying conditions. This compensates for certain shortcomings of previous models and further improves the risk assessment system of hazmat transportation under time-varying conditions.

Therefore, this paper not only promotes the development of the risk analysis theory, technology and methods of hazmat transportation to a certain extent, but also provides support for the scientific decision-making process in regard to hazmat road transportation locations and path optimization. More specifically, the results retrieved from the proposed model could be used as basics for hazmat transportation location and path optimization to help decision makers scientifically formulate decisions regarding hazmat transportation location and path optimization.

In addition, through the case study, it was clearly revealed that in practice, controlling certain parameters in the transportation risk assessment model could reduce the probabilities and consequences of hazmat road transportation accidents. We should also avoid road segments with a high risk of catastrophic accident consequences to improve the safety of hazmat transportation.

Several directions of research should be explored in the future. This paper should be applied to a larger case to test and improve the theoretical methods, and risk assessment of hazmat multimodal transportation under time-varying conditions should be further studied to more closely reflect reality.

## Figures and Tables

**Table 1:** Weighted average truck accident probability (accidents per million vehicle/km) in three states of the United States.

Region	Road Type	$P_{ij}^{A}$
City	Two-lane road	5.38
	Undivided multilane road	8.65
	Divided multilane road	7.75
	Cycle lane	6.03
	Expressway	1.35

**Table 2:** Leakage accident probability under certain accidents.

Accident Type		$P_{ij}^{B}$
Single-vehicle noncollision accident	Leaves the road	0.331
	Overturned on the road	0.375
	Other noncollision accident	0.169
Single-vehicle collision accident	Collision with stopped vehicle	0.031
	Collision with a train	0.455
	Collision with a nonmotorized vehicle	0.015
	Collision with fixed objects	0.129
	Other collision accident	0.059

**Table 3:** Classification of the AEGL injury threshold levels.

Grade	Definition
AEGL-1	The airborne concentration of a substance above which it is predicted that the general population, including susceptible individuals, could experience notable discomfort, irritation, or certain asymptomatic nonsensory effects. However, the effects are not disabling and are transient and reversible upon cessation of exposure.
AEGL-2	The airborne concentration of a substance above which it is predicted that the general population, including susceptible individuals, could experience irreversible or other serious, long-lasting adverse health effects or an impaired ability to escape.
AEGL-3	The airborne concentration of a substance above which it is predicted that the general population, including susceptible individuals, could experience life-threatening health effects or death.

Note: the information in Table 3 refers to [44].

## Data Availability

The data presented in this study are available upon request from the authors.
